# Towards the Use of Waste Pig Fat as a Novel Potential Bio-Based Rejuvenator for Recycled Asphalt Pavement

**DOI:** 10.3390/ma13041002

**Published:** 2020-02-23

**Authors:** Nader Nciri, Taesub Shin, Namho Kim, Arnaud Caron, Hanen Ben Ismail, Namjun Cho

**Affiliations:** 1 School of Energy Materials⋅Chemical Engineering, Korea University of Technology & Education, 1600 Chungjeol-ro, Byeongcheon-myeon, Dongnam-gu, Cheonan, Chungnam 31253, Korea; 2 Department of Chemistry, Seoul National University, 1 Gwanak-ro, Gwanak-gu, Seoul 08826, Korea; 3 Department of Animal Resources, Fisheries, and Food Technology, National Institute of Agronomy of Tunisia, 43 Avenue Charles Nicolle, 1082 El Mahrajène, Tunis, Tunisia; hanenbenismail90@gmail.com; 4 School of Industrial Design⋅Architectural Engineering, Korea University of Technology & Education, 1600 Chungjeol-ro, Byeongcheon-myeon, Dongnam-gu, Cheonan, Chungnam 31253, Korea

**Keywords:** reclaimed asphalt pavement (RAP) binder, waste fig fats, bio-based rejuvenator, SARA generic fractions, morphology, topography, empirical tests, rutting resistance, fatigue cracking resistance, temperature susceptibility, thermal properties

## Abstract

This article presents a novel potential bio-based rejuvenator derived from waste pig fat (WPF) for use in recycled asphalt applications. To achieve this purpose, the impact of different doses waste pig fat (e.g., 0, 3, 6, and 9 wt.% WPF) on the reclaimed asphalt pavement binder (RAP-B) performance is investigated. The unmodified and WPF-modified asphalts are characterized by means of Fourier-transform infrared spectroscopy (FT-IR), thin-layer chromatography–flame ionization detection (TLC-FID), scanning electron microscopy (SEM), atomic force microscopy (AFM), thermogravimetric analysis (TGA), and differential scanning calorimetry (DSC). Physico-rheological properties of asphalt blends are assessed through Brookfield viscometer, softening point, penetration, and dynamic shear rheometer (DSR) tests. TLC-FID data highlighted that incremental WPF addition into RAP-B restored its original balance maltenes-to-asphaltenes ratio; finding which was supported by FT-IR analysis. SEM disclosed that WPF has a great compatibility with the aged asphalt. AFM observations showed that grease treatment induced a decline in surface roughness (i.e., bee structures) and a rise in friction force (i.e., para-phase dimension) of RAP binder. TGA/DSC studies revealed that the bio-modifier not only possesses an excellent thermal stability but also can substantially enhance the binder low-temperature performance. Empirical and DSR tests demonstrated that WPF improved the low-temperature performance grade of RAP-B, reduced its mixing and compaction temperatures, and noticeably boosted its fatigue cracking resistance. The rejuvenation of aged asphalt employing WPF is feasible and can be an ideal approach to recycle both of RAP and waste pig fats.

## 1. Introduction

The use of processed reclaimed asphalt pavement (RAP) in paving mixtures has been common practice in the Republic of Korea. However, provincial transportation agencies are often unwilling to adopt higher RAP levels, typically larger than 20 wt.% by weight of total mixture. Incorporating substantial amounts of RAP into new paving mixtures constitutes a critical concern, because the resultant blends may be vulnerable to adhesion and cohesion distress as well as cracking failures during the service life of the road pavement [1]. This is due primarily to the oxidative aging of the asphalt binder coating the aggregates enclosed in the RAP material. In the oxidation process, atmospheric oxygen interacts with the aromatics and resins to yield asphaltenes. Accordingly, this gives rise to a variety of polar groups (i.e., higher molecular weight fraction) at the expense of the lower molecular weight constituents, which in turn will contribute to an increase in the asphalt viscosity [2]. Moreover, the asphalt becomes rheologically unstable owing to the discontinuity that occurs between the saturated compounds and the remaining chemical species (i.e., aromatics, resins, and asphaltenes) [3]. This instability eventually provokes a lack of cohesion within the binder, which may lead to cracking. Oxidation induces drastic changes in the molecular structure of the asphalt, resulting thereby in a stiffening of the bituminous material, a loss in its ductility, and a reduction in its ability to relieve stress. Therefore, the pavement is more susceptible to stress accumulation, generally accompanied with thermally induced-stress caused by cooling [3]. The degree of oxidation is chiefly governed by temperature and is intense at the high temperatures employed for mixing, laying, and compacting of bituminous materials [4]. Using larger volumes of RAP in new paving mixtures will aggravate the aforementioned problems, as elevated quantities of aged RAP binder will be ultimately subjected to extra aging during production, construction, and service [5].

A common approach to alleviate the undesirable effects of the aged binder on the performance of RAP-contained mixtures consists in using a softer virgin binder, when greater than normal RAP contents of 15–20 wt.% are utilized [6]. Notwithstanding that fresh soft binders have been used in an attempt to compensate for the stiffness of aged RAP binders, numerous research projects have revealed that asphalt rejuvenating agents can permit vastly higher percentages of RAP to be incorporated in asphalt mixes than softer binders alone [7,8,9]. However, it is of utmost importance to carefully select the appropriate rejuvenator to achieve the desired performance. Asphalt rejuvenators are produced to recover the physical and rheological properties, such as viscoelastic behavior of RAP binders by rapidly diffusing into them, restoring their colloidal structure, and reconstituting their chemical components [10]. Softening additives have been broadly used in pavement preservation to revive hard surface layers by percolating into the pavement, replenishing the oils lost and mobilizing the aged binder to balance the maltenes (i.e., saturates, aromatics, and resins)-to-asphaltenes ratio [11]. The impact of various types of rejuvenators on the bitumen performance has been investigated by several engineers and scientists. Empirical binder tests including softening point, needle penetration, and dynamic viscosity revealed the great potentiality of rejuvenators to effectively decrease the viscosity and stiffness of RAP binder in question, thereby enhancing its low-temperature cracking resistance and improving its workability during construction [12]. Different types and dosages of rejuvenating agents—such as aromatic extract [13], waste engine oil [14], and refined tall oil [13]—can be adopted to reach the optimal correlation with the amount and type of RAP binder. The majority of current rejuvenators are petroleum-based products and suffer from increasing demand and correspondingly cost, while supplies are shrinking. A source of non-petroleum based materials is urgently needed in the asphalt industry. 

Fats, oils, and greases (FOGs) are widely used in the agro-food industry, and their waste impact is becoming a fast-growing concern in pollution control. The main issue of FOGs in waste management is related particularly to their insolubility in water. This feature creates serious problems to the water and sewage treatment plants throughout the country [15]. Currently, there are no feasible, sustainable, and suitable means for the disposal of FOG wastes from major food processing plants, food service establishments, domestic properties, and several other entities. Burial in landfills is an inviable and irresponsible strategy. It is expected that by the year 2050, there will not be enough landfill space to bury half of Korean municipal wastes [16]. Extensive research efforts have been devoted to the area of oil/fat conversion into diesel fuels [17,18,19]. Nevertheless, those ventures have been hampered by conversion difficulties and high production costs. 

One ideal approach to reduce waste fats and oils could be through incorporating them in reclaimed asphalt pavement as bio-based rejuvenating agents. For instance, it has been found that waste cooking oils (WCOs) can prolong the pavement service life [20]. The addition of WCO to the recycled bitumen has been shown to improve the engineering performance of the resulting mixture; as the fatty acids (saturated and unsaturated fatty acids) existing in WCO have been shown to act as cohesive and adhesive agents, reducing the high viscosity of the aged binders, enabling homogenous mixing, and diminishing surface tension with new pavement materials [21]. Previous assessments conducted on binder performance [21,22] demonstrated that 3–4 wt.% WCO could recover the original binder properties by generating a high penetration value from the old binder. Also, it has been stated that vegetable oils can lower the stiffness of the old asphalt effectively and greatly improve its fatigue cracking resistance [23]. Although there is a large body of research literature on the use of waste vegetable oils for regenerating old bitumen, there have been limited studies examining the use of waste animal fats as asphalt rejuvenators. 

Accordingly, the primary purpose of this research effort is to create an eco-innovative asphalt binder through the substitution of standard asphalt mixtures by a combination of reclaimed asphalt pavement binder (RAP-B) and waste pig fat (WPF) as bio-based rejuvenator. The current investigation is intended to establish a new concept of asphalt pavement structures with ecologically oriented attributes, significantly reducing waste disposal problems, while achieving a level of long-term performance comparable or greater than that of conventional pavement structures. Eco-friendliness, energy efficiency, cost effectiveness, and sustainability are major drivers behind this project. It is hoped that this effort will provide sufficiently detailed information to establish the benefits, drawbacks, and overall feasibility of using waste animal fats as softening bioadditives in recycled asphalt applications.

## 2. Materials and Methods

### 2.1. Waste Pig Fat (WPF) Preparation

**Figure 1:**
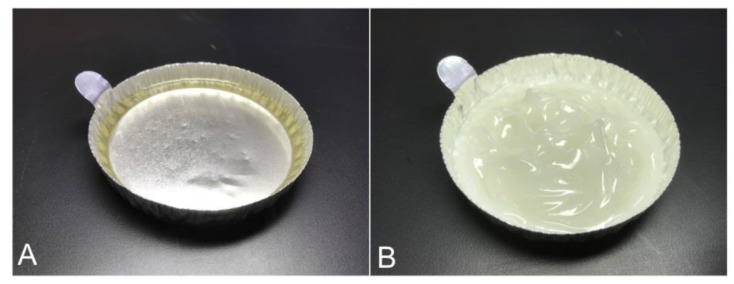
Appearance of waste pig fat (WPF) at 60 °C (**A**) and 25 °C (**B**).

WPF shown in Figure 1 is an appealing raw material, as it serves as a cheap source and its utilization serves as an environmental cleaner. For this study, WPF freshly collected from a local butchery in South Korea is mainly consisting of fat and residual skin and meat. The adipose tissues of pigs were finely cut into small pieces and mixed. Then, the diced pork fats were slowly heated in a 1 L-stainless steel pot at 110 °C with stirring under atmospheric pressure. After the tissues had been heated and stirred for 1 h, the rendered fats were filtered through a double-folded muslin cloth to remove the insoluble impurities such as meat and bone particles. To ensure effective moisture removal, the samples were treated with small proportions of anhydrous sodium sulphate (Na_2_SO_4_, 98.00%). Finally, the treated fat was passed through a Whatman no. 2 filter paper cone, stored in loosely capped glass jars and kept in the fridge at 4 °C to prevent oxidation during the experimental period. Before being used for analysis, the animal fat was thawed at 60 °C until it melted, and added to the aged asphalt (RAP-B) at the desired dose (e.g., 0, 3, 6, and 9 wt.%). The physicochemical characteristics of WPF, given in Table 1, were determined with the assistance of the Department of Animal Resources, Fisheries, and Food Technology, National Institute of Agronomy of Tunisia, El Mahrajène, Tunis, Tunisia.

### 2.2. Preparation of Waste Pig Fat (WPF)-Asphalt Blends

The RAP material used in this investigation was supplied from a Korean asphalt contractor (Korea Institute of Civil Engineering & Building Technology, Goyang, Korea). The RAP was from a single source and from a well-managed stockpile. Before being delivered to the laboratory in Cheonan, Korea, it was sampled and tested for determining the performance grade (PG) of the binder in the RAP according to ASTM D946 [24]. The RAP binder was carefully extracted in accordance with AASHTO T 164 standard test method (A) [25], using the centrifuge apparatus and trichloroethylene as a solvent. Once the RAP-B is extracted from the RAP aggregates, it was recovered using the rotary evaporator following ASTM D5404 testing procedure [26]. This step allows the binder to be separated by solvent evaporation. The recovered RAP-B was graded according to the Superpave performance grading (PG) system by testing the RAP binder as original (unaged), after short-term aging through the rolling thin film oven (RTFO) [27], and after long-term aging through the pressure aging vessel (PAV) [28]. The RTFO is designed to simulate the aging process of asphalt during plant mixing, transportation, and paving, while the PAV is used to simulate aging during in-service life. The extracted RAP aggregates were sieved to determine the gradation in accordance with ASSHTO T 30 standard test method [29] for mechanical size analysis of extracted aggregates (data not shown). The RAP-B had recovered 4.60 wt.% by weight of RAP mix. The physicochemical properties of the RAP binder are reported in Table 2. The RAP-B was used to generate three different rejuvenated asphalt samples by adding WPF at different fractions. All modified asphalts were produced in the laboratory using a L5M-A mixer (Silverson, v, USA), operating at a rotation speed of 3000 rpm at 180 °C. Initially, 600 g of asphalt contained in a 1000 mL stainless steel can were preheated to fully fluid condition. Up on reaching 175 °C, the melted WPF was slowly poured to the asphalt at various doses of 0, 3, 6, or 9 wt.% by weight of bitumen. After reaching 180 °C, stirring was continued at this temperature for 2 h to guarantee homogeneous blends were achieved. Afterwards, the hot asphalt binder was removed from the can and distributed in small containers. The blend was cooled to room temperature (ca. 25 °C), sealed with aluminum foil and stored for subsequent experiments.

### 2.3. Fourier-Transform Infrared Spectroscopy (FT-IR)

The FT-IR spectroscopy was performed on a Hyperion 3000 FT-IR Spectrometer (Bruker Optics, Ettlingen, Germany) to probe the changes in functional groups of RAP-B after treatment with WPF. The samples were analyzed using thin disk of the sample mixed with potassium bromide (i.e., KBr) in the range of 600 to 4000 cm^−1^, with 32 scans.

### 2.4. Thin-Layer Chromatography–Flame Ionization Detection (TLC-FID)

To explore the impact of WPF addition on the composition of RAP-B, unmodified and modified bituminous samples were chemically characterized. In thin-layer chromatography with flame ionization detection, TLC-FID (Iatroscan analyzer, Iatron, Tokyo, Japan), 2% (w/v) solutions of binders were prepared in dichloromethane solvent, and 1 µL sample solution spotted on chromarods using a spotter. The separation of asphalt binder into four generic fractions i.e., saturates, aromatics, resins, and asphaltenes, was carried out with a three-stage process using *n*-heptane, toluene, and dichloromethane/methanol (95/5 by volume), respectively. The saturates, aromatics, resins, and asphaltenes were eluted stepwise, and the most polar asphaltenes remained at their original place on the chromarod. Finally, the chromarod was dried at 80 °C for 1 min in the FID, the individual separated zones were ionized in the hydrogen flame, and the ionized currents were recorded. The applied scan rate was 30 s/scan, and the air and hydrogen flows were 2000 mL/min and 170 mL/min, respectively.

### 2.5. Scanning Electron Microscopy (SEM)

Scanning electron microscopy (SEM) (JSM-6010LA, JEOL Ltd., Tokyo, Japan) was utilized to examine the microstructure and morphology of RAP-B with 0, 3, 6, or 9 wt.% of WPF. The specimens were cryogenically fractured in liquid nitrogen to guarantee a sharp brittle fracture, and were successively sputter-coated with 12 nm gold to render them conductive prior to SEM observation. For optimal BSE (backscattered electron) collection, the working distance used was 10 mm from the final lens. Previous researchers found that SEM is highly preferable because it provides a clear view of a material in the raw state [30,31].

### 2.6. Atomic Force Microscopy (AFM)

Atomic force microscopy (AFM, XE-100, Park Systems, Suwon, South Korea) was applied to investigate the effect of WPF on the surface microstructure and micromechanical behavior (e.g., friction) of RAP-B. A hot liquid drop of asphalt at 140 °C was carefully brought onto a glass microscope slide (25 × 75 mm) and subsequently kept covered inside a Petri dish to prevent dust pick-up. Samples were annealed at ambient temperature, for a minimum of 24 h before imaging. AFM measurements were recorded by contact mode AFM using a soft single-crystalline silicon cantilever (Type: ppp-contsc, NanoSensors, Switzerland). Prior to imaging bitumen, we calibrated the sensitivity of the photodiode by recording a force-distance curve on a non-compliant nanocrystalline diamond thin-film. Subsequently, the normal and lateral stiffness of the photodiode were determined by the thermal noise analysis (C_n_ = 0.02 N/m and C_l_ = 3.97 N/m) [32]. Both topography and lateral force images were recorded. AFM images were recorded at various locations on the bituminous sample surface with a normal force-value *F_n_* = 0 nN. We calculated the friction force maps according to *F_f_* = (*F_l, fwd_ − F_l, bwd_*)/2, where *F_l_*_, *fwd*_ and *F_l_*_, *bwd*_ are the lateral force signals recorded in the forward and backward direction, respectively. The unit of the lateral force signals were converted from the lateral voltage of the photodiode into unit of force according to *F_l_* = *S⋅C_l_⋅V_l_⋅*(*3h/2L*), where *h* is the tip height, *L* is the length of the cantilever, *S* is the sensitivity of the photodiode and *V_l_* is the lateral voltage of the photodiode.

### 2.7. Thermogravimetric Analysis (TGA/DTGA)

The thermal properties of WPF, unmodified and WPF-modified asphalts were analyzed using a thermogravimetric analyzer (TGA Q500, TA Instruments, New Castle, DE, USA). About 10 mg sample was heated from room temperature to 1000 °C with a heating rate of 10 °C/min under 150 mL/min N_2_ flow.

### 2.8. Differential Scanning Calorimetry (DSC)

The differential scanning calorimetry (DSC) analysis was conducted using a PerkinElmer 8000 DSC (PerkinElmer, Waltham, MA, USA). Approximately 15–20 mg of bitumen sample was weighted in an open pan and placed in the DSC cell under nitrogen blanket. The sample was heated to 50 °C and remained for about 10 min to ensure stabilized initial reading. Afterwards, the sample was cooled down to −90 °C at 20 °C/min, subsequent to which the heating rate was set at 20 °C/min to reach 150 °C. The cooling and heating processes were designed to erase all traces of the thermal history of asphaltic samples. Once completing the first scan, the sample was quickly cooled down from 150 °C to −90 °C and held for about 10 min before being reheated to 150 °C at a heating rate of 20 °C/min. The DSC thermogram recorded during this second heating scan is designated as the second scan from which the calorimetric parameters were extracted.

### 2.9. Conventional Binder Tests (Penetration, Softening Point, and Viscosity)

According to ASTM standards, some conventional tests were conducted on the sample such as penetration, softening point, and viscosity using the Brookfield Model DV-III (Brookfield, Middleboro, MA, USA). The penetration test was carried out according to ASTM D5 [33] and is used as a measure of consistency. The softening point was measured based on ASTM D36 [34]. The standard rotational viscometer test was performed based on ASTM D4402 [35]. The viscosity was determined from the amount of torque required to rotate a cylindrical spindle 27 (SC4-27), which has a shear rate of 6.8 s^−1^, at a constant speed of 20 rpm, while submerged in the sample chamber filled with 10 mL of binder at 135 °C.

### 2.10. Temperature Susceptibility

The effect of WPF addition on the temperature susceptibility of reclaimed asphalt pavement binder (RAP-B) was investigated by determining penetration index (PI) and penetration viscosity number (PVN). The higher the PI and PVN values of asphalt binder, the lower is its temperature susceptibility. For PI, the temperature susceptibility for the binders is measured by calculating the PI using the penetration at 25 °C and softening point data:

Penetration index (PI) can be calculated precisely from Equation (1), as follows [36]

(1) $$\mathrm{PI}=\frac{1952\;-\;500\log\;\left(\mathrm{Pen}\;25\right)\;-\;20\;\times \;\mathrm{SP}\;}{50\log\;\left(\mathrm{Pen}25\right)\;-\;\mathrm{SP}\;-\;120}\;$$

where Pen is the penetration test (0.1 mm) at 25 °C and SP is the softening point (°C).

The penetration-viscosity number (PVN) is calculated from Equation (2) based on penetration (0.1 mm) at 25 °C and viscosity (cSt) at 135 °C as [36]

(2) $$\mathrm{PVN}=-1.5\;\frac{\log L-\log X}{\log L-\log M}$$

X: viscosity in centistokes at 135 °C associated with the penetration at 25 °C of the paving bitumen for which the PVN value is needed. L: for the same penetration at 25 °C, the corresponding viscosity in centistokes at 135 °C for which PVN = 0.0, or 4.24800 – 0.79674 log (Pen). M: for the same penetration at 135 °C, the corresponding viscosity in centistokes at 135 °C for which PVN = −1.5, or 3.46289 – 0.61094 log (Pen). P: penetration at 25 °C of the paving bitumen for which PVN is needed.

### 2.11. Dynamic Shear Rheometer (DSR) Test

According to ASTM D7175 [37], a dynamic shear rheometer (DSR, MCR101, Anton Paar, Hanover, VA, USA) was employed to characterize the viscous and elastic behavior of bitumen. DSR test allows to measure the rheological properties of asphalt binder in terms of dynamic shear modulus (i.e., stiffness), G*, and the phase angle, δ (i.e., elasticity). In the SHRP asphalt binder specification, G*/sin δ relates to permanent deformation (i.e., rutting) and the parameter G*⋅sin δ relates to fatigue cracking. The loading frequency was 1.59 Hz (10 rad s^−1^). In all tests, the 8 mm-diameter plate and 2 mm-gap were used when test temperatures were lower than 40 °C; otherwise, parallel plates with 25 mm-diameter and 1 mm-gap were used. All test results shown in this paper represent the average of three replicates.

## 3. Results and Discussion

### 3.1. Fourier-Transform Infrared Spectroscopy (FT-IR)

**Figure 2:**
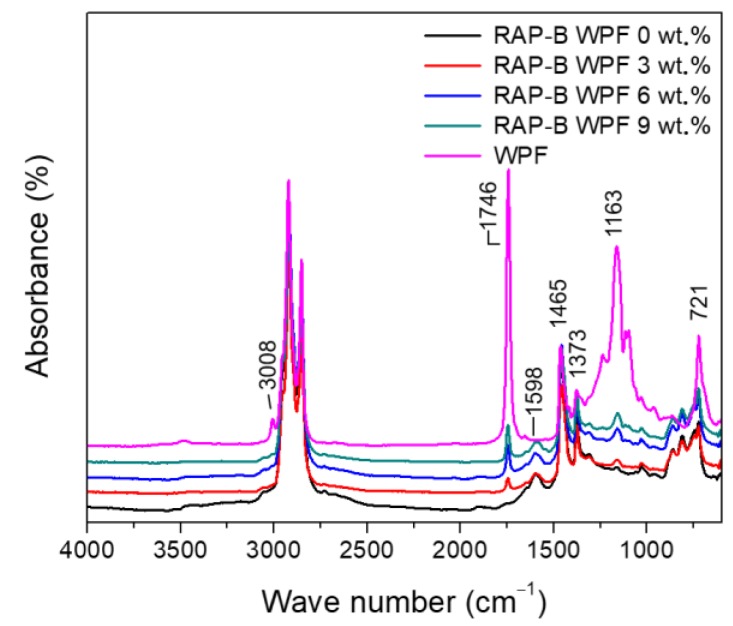
FT-IR spectra of WPF, RAP-B modified with 0, 3, 6, and 9 wt.% of WPF.

FT-IR spectroscopy was employed to elucidate the influence of WPF on the functional groups, molecular structures and composition of reclaimed asphalt pavement binder (RAP-B). Figure 2 shows the FT-IR spectra of WPF, RAP-B unmodified and modified with various fractions of WPF in the range of 4000–650 cm^−1^. In the IR spectrum of WPF, the wavenumber of 3008 cm^−1^ comes from the cis-olefinic =C-H stretching vibration which can be used as an indicative of unsaturation degree. The greater the unsaturation degree of fat, the higher the peak intensity in that position. WPF can easily be discerned from the bands and shoulders that are associated to specific composition, length, functional groups, and degree of unsaturation [38]. The strong peaks within 2925–2851 cm^−1^ region are typical C–H stretching vibrations in aliphatic chains. The peak at 1465 cm^−1^ is attributed to CH_2_ bending vibration with the contribution from asymmetric bending of CH_3_ and the peak at 1373 cm^−1^ is ascribed to symmetric bending of CH_3_. The peaks at 1746 cm^−1^ and 1163 cm^−1^ stretching vibration of C=O and C-O of fatty acid ester, respectively. The peak at 721 cm^−1^ is due to rocking mode of CH_2_, so called long-chain band [39].

In the IR spectrum of RAP-B, the relative peak height of 1373 cm^−1^ vs. 1465 cm^−1^ is much higher than that of WPF, which indicates RAP-B contains many -CH_3_ groups in branched hydrocarbons. The characteristic absorption peak around 1598 cm^−1^ refers to the aromatic ring stretching vibrations of aromatics and polyaromatic hydrocarbons. This band is obviously absent in the WPF spectrum; however, its intensity is slightly diminished after the incremental WPF introduction into the RAP-B, reflecting a decrease in the asphaltenes content. The sharp peak about 1746 cm^−1^ is consistently increased as the amount of WPF added into RAP-B is increased. This 1746 cm^−1^ peak corresponding to the carbonyl C=O stretching vibrations of fatty acid ester in WPF. The carbonyl peak is absent in the straight asphalt spectrum and its intensity increased linearly following the progressive introduction of grease into the RAP-B, indicating an increase in the long chain maltenes content which is responsible for making the binder softer. To sum up, the bio-based additive has a significant effect on the chemical structure and composition of aged binder which will ultimately affect the mix performance.

### 3.2. Thin-Layer Chromatography-Flame Ionization Detection (TLC-FID)

**Figure 3:**
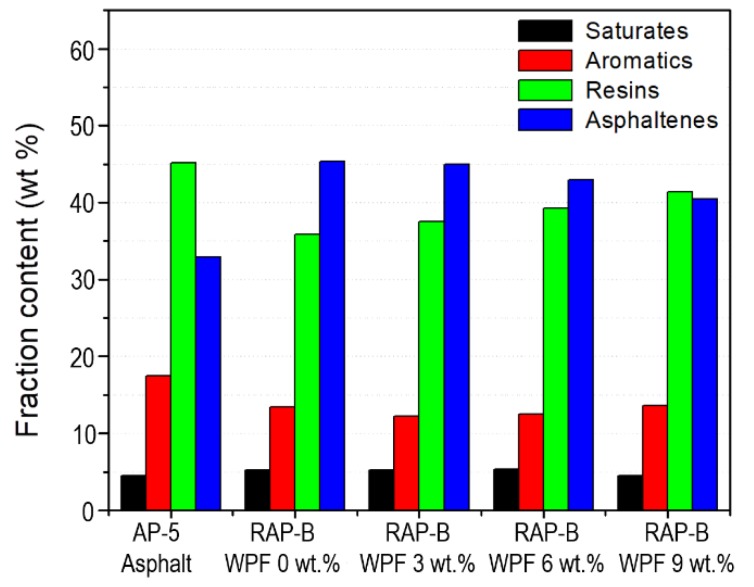
Effect of different concentrations (0, 3, 6, 9 wt.%) of WPF on the SARA fractions of RAP-B.

To shed some new light on the mechanisms by which WPF affects the chemical composition of RAP-B, TLC-FID technique was conducted on the reference fresh AP-5 asphalt (PG 50–60) and RAP-B rejuvenated with various portions of WPF. Figure 3 shows a typical Iatroscan-histogram for the neat RAP-B, which displays four distinct bands of saturates (5.09 wt.%), aromatics (12.94 wt.%), resins (38.53 wt.%), and asphaltenes (43.44 wt.%). This composition is commonly known as SARA fractions. As seen from Figure 3, an incremental addition of WPF, which is perceived as 100 wt.% resins, into the base bitumen (RAP-B) reduced asphaltenes content and at the same time increased the aromatics and resins content of the maltene phase. Nonetheless, the content of saturates remains almost unaffected (revealing an obviously lower chemical reactivity). This indicates that there may be some chemical reactions happening following the grease treatment. WPF acts as an active peptizing agent that can be adsorbed on asphaltene molecules and enhances greatly their dispersion in an oily liquid medium (i.e., maltenes). Through reducing the high content of asphaltenes and increasing the resins and aromatics content, WPF seemingly possesses a regenerative effect on RAP-B; therefore, it can possibly be used as a potential rejuvenator agent to restore the original chemical balance of aged bitumens.

### 3.3. Scanning Electron Microscopy (SEM)

**Figure 4:**
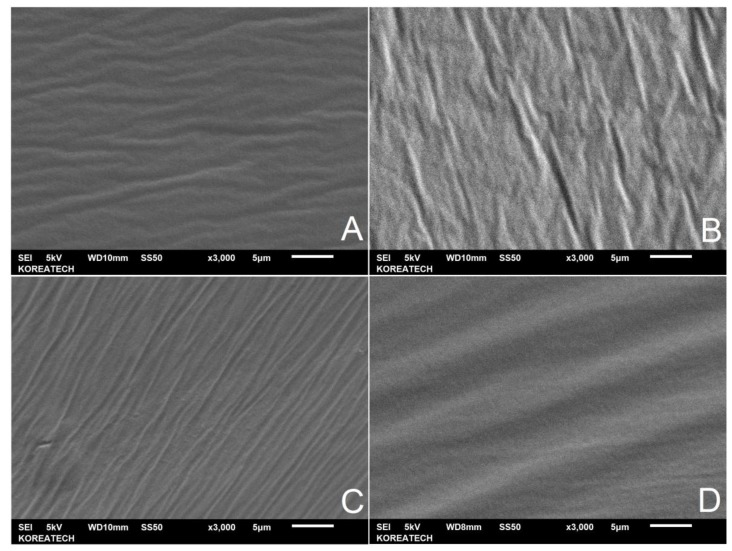
SEM images of unmodified and WPF-modified RAP-B taken at × 3000 magnification. (**A**) RAP-B WPF 0 wt.%; (**B**) RAP-B WPF 3 wt.%; (**C**) RAP-B WPF 6 wt.%; (**D**) RAP-B WPF 9 wt.%.

The morphological study for the RAP binder was carried out with SEM to understand and observe the morphology, surface texture, and compatibility of waste pig fat (WPF) with the aged asphalt (RAP-B). The fracture surface images of RAP-B and RAP-B modified with different fractions of WPF are portrayed in Figure 4. As shown in Figure 4A, the RAP-B exhibits a flat and smooth fracture surface without any gully and layer structure. Conversely, many hilly and gully-like veins are dispersed in the Figure 4C,D which are distinctly different from that of RAP-B. On the other side, no visible WPF and interfaces between rejuvenator and asphalt are observed in Figure 4, indicating the superior compatibility between asphalt binder and WPF. Relatively, scabrous and multilayer structure morphology is also detected for the RAP-B containing 9 wt.% of WPF (Figure 4D). However, the phase separation does not appear even the WPF content reaches 9 wt.%, implying thereby the great miscibility of bio-additive and bitumen. The great compatibility between WPF and asphalt is expected to enhance the durability and reliability of asphalt/WPF prepared blends. Overall, increasing the dosage rate of rejuvenator resulted in a much smoother fracture surface.

### 3.4. Atomic Force Microscopy (AFM)

**Figure 5:**
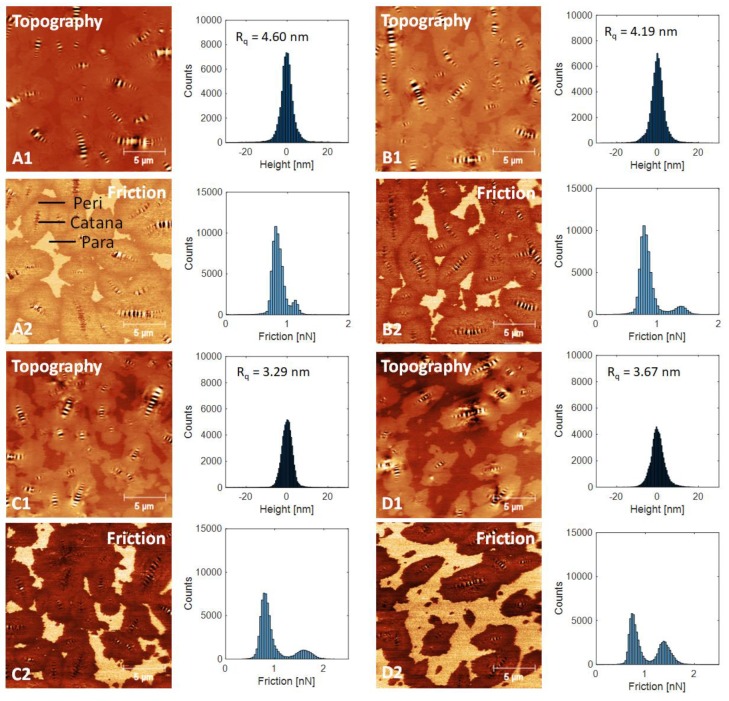
Flattened topography (denoted with number 1) and friction (denoted with number 2) images show the impact of WPF on asphalt morphology and friction. On the right-hand side of each AFM image, we further present the corresponding histogram. (**A**) RAP-B WPF 0 wt.%; (**B**), RAP-B WPF 3 wt.%; (**C**) RAP-B WPF 6 wt.%; (**D**) RAP-B WPF 9 wt.%.

Contact mode AFM measurements were carried out to analyze the impact of bio-based rejuvenator on the surface microstructure and micromechanical behavior of asphalt binder. The AFM topographic images (deposited on a glass microscope slide) of RAP-B mixed with various doses (0, 3, 6, and 9 wt.%) of WPF are shown in Figure 5(A1, B1, C1, and D1). The corresponding friction images are shown in Figure 5(A2, B2, C2, and D2), respectively. The images reveal a homogenous matrix in which another phase is dispersed. Both phases have distinct material properties, that result in a contrast difference in the friction force images. This leads to the appearance of lighter (elliptic domains with larger friction force) and darker (matrix with lower friction force) regions, as can be verified from Figure 5.

The dispersed phase shows a succession of pale and dark lines often being referred to as “bees” or “bee-like structures” [40], and its corresponding topographic profile is shown in Figure 5. The dispersed phase is also known as the ‘catana’ or catanic phase, from the Greek ‘cata’, high to low, and ‘ana’, low to high [40]. Figure 5(A2) reveals not only the catana-phase, but also other phases. Immediately around the catana-phase exists a dark-looking phase, separated here and there by another phase of a lighter shade. These two phases are called ‘peri-‘ and ‘para-‘ phases, respectively (from the Greek ‘peri’, around; and ‘para’, neighboring) [40]. Each para-phase also contains small quasi spherical domains termed the ‘sal’ phase (‘sal’, Latin for salt) [40]. This phase is finely dispersed in the para-phase of bitumen, but it was absent from the bituminous specimens investigated here, although the catana-, peri-, and para-phases were clearly visible. All binders clearly revealed enriched microstructural features.

Blending the RAP-B with WPF was actually initiated with the rearrangement of the bee structures by increasing their numbers (particularly smaller ones) and decreasing their sizes. The bee structures are most probably correlated with the saturate components (crystalline paraffin wax, predominantly linear *n*-alkanes) or microcrystalline wax (naphthene and *iso*-alkanes) of RAP binder [41,42]. On the other hand, the tiny bee structures observed on the surface of WPF-modified asphalt samples are most likely attributed to the free fatty acids and their ester derivatives which contain few crystallizing waxes. It can be seen also that the dimension of peri-phase decreased, meanwhile the dimension of para-phase increased. At this level, no possible correlation can be identified between the AFM morphology and the four generic fractions of asphalt binder (saturates, aromatics, resins, and asphaltenes) and more in-depth studies are needed.

Surface roughness data are further portrayed in Figure 5. Higher roughness value usually stands for more and/or large sized bee structures. It is proved again that WPF addition can impede the growth of bee structure. Asphalt samples with higher content of WPF (6 and 9 wt.%) usually show lower roughness values. The roughness feature of the surface microstructure of the binder is considered as one of the major factors that will affect the adhesion with the aggregates in the bituminous mixtures. It is expected that suitable grease shots (less than 3 wt.%) will enhance the mechanical strength performance of HMA by improving its coatability potential; hence, preventing the mix from moisture damage and raveling.

Figure 5 illustrates also the friction force as histograms of the RAP-B before and after treatment with various concentrations of WPF (3, 6, and 9 wt.%) obtained with the lateral force mode of the AFM. The bright regions account for areas where the friction forces are stronger, whereas the darker ones correspond to weaker friction forces. The clear zones are due in shape and size to the para-phase structures, demonstrating that those structures are mainly involved in sustaining the shear stress of the binder mixture and their relative amount and spatial configuration would ultimately influence the final mechanical behavior of the resulting system. To simplify the visualization of the obtained data, these maps were converted into several histograms for the measured friction (in terms of friction force in nN) on the surface. All the histograms of asphaltic blends are composed of two distinct bands. The first band, located at around 0.75 nN, corresponds to the darker zones in the topograph and is referred to the peri-phase, while the second band (friction value higher than 1 nN) is assigned to the brighter para-phase domains.

### 3.5. Conventional Physical Properties (Penetration, Softening Point, and Viscosity)

**Figure 6:**
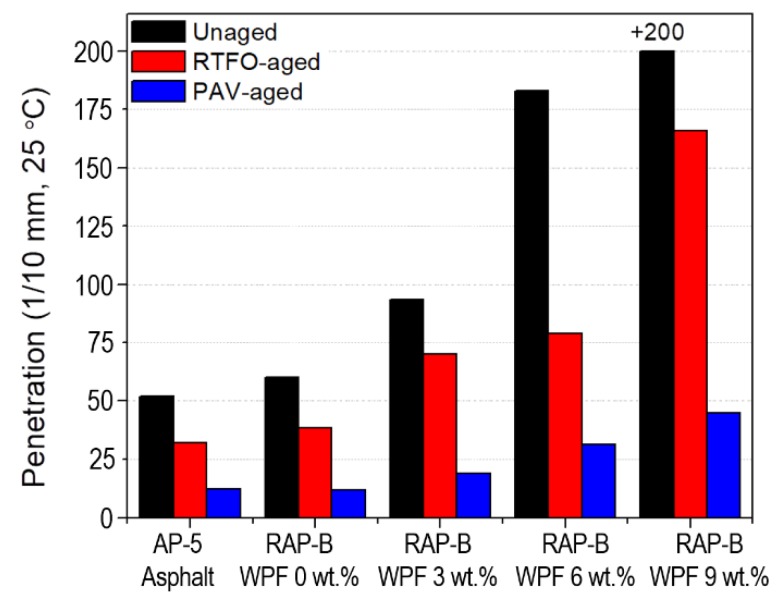
Effect of different concentrations (0, 3, 6, 9 wt.%) of WPF on the penetration of RAP-B before and after RTFO and PAV aging.

**Figure 7:**
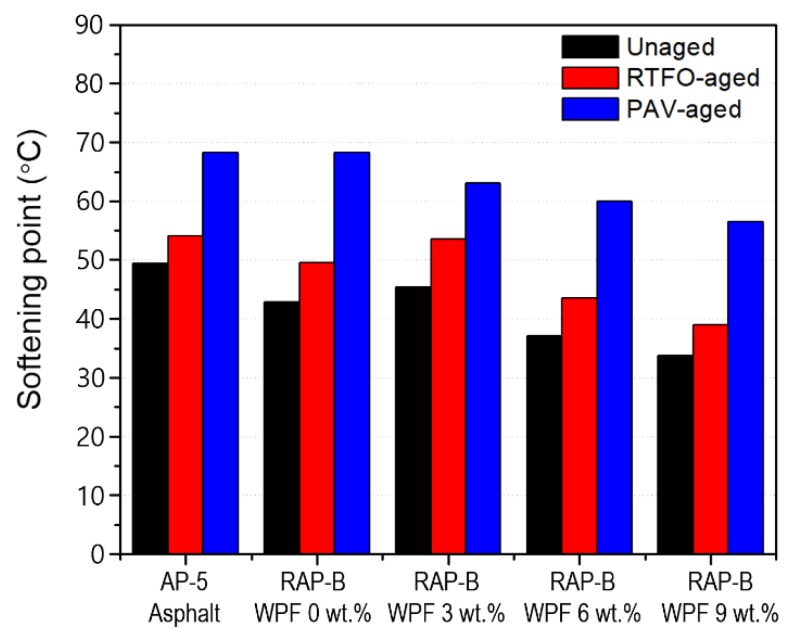
Effect of different concentrations (0, 3, 6, 9 wt.%) of WPF on the softening point of RAP-B before and after RTFO and PAV aging.

**Figure 8:**
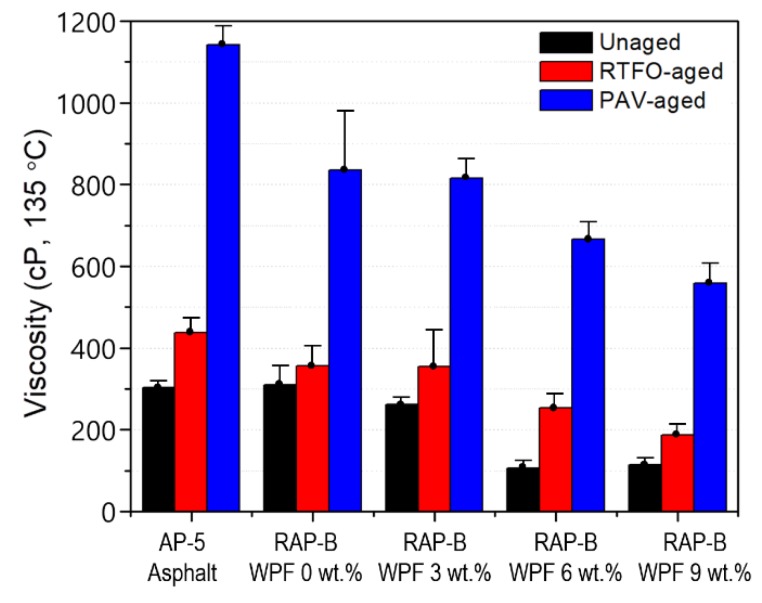
Effect of different concentrations (0, 3, 6, 9 wt.%) of WPF on the viscosity of RAP-B before and after RTFO and PAV aging.

The penetration, softening point, viscosity data of unaged, and RTFO- and PAV-aged asphalt samples containing different fractions of WPF are shown in Figure 6, Figure 7 and Figure 8, respectively. In addition to RAP-B, a Superpave performance graded binder, AP-5 (PG 50–60) was used as a reference.

As visualized in Figure 6, the RAP-B shows a PG 60–70 behavior, all unaged and aged-bitumen specimens display the increased penetration values with increase in WPF content. The decrease in binder hardness can be attributed mainly to the rejuvenating effect caused by the addition of WPF to the non-aged or aged asphalts. The results indicate that the addition of up 3 wt.% WPF can favorably soften the asphalt by reducing its consistency and restoring its flexibility–plasticity. On the other side, blending the RAP binder with higher concentrations of grease (particularly 6 or 9 wt.%) is expected to generate fluid asphalt, which will result in pavements of lower stability at all temperatures above 25 °C. Therefore, the amount of WPF incorporated should be controlled according to demands.

The softening point can mirror the high temperature stability of asphalt. Referring to Figure 7, the softening point temperature shows an overall decline trend with increasing the WPF dosages, thus indicating an identical drop in hardness or stiffness of unaged and short or long-term aged asphalt samples, as witnessed in the penetrations (Figure 6). Interestingly, it was found that 3 wt.% of bio-additive can practically restore the thermoplastic property of RAP-B and bring it back to its original state, with respect to AP-5 asphalt.

The viscosity of bitumen refers to its ability to resist shear deformation under external forces and at 135 °C; the viscosity is typically used to represent the compacting temperature of binder mixture. Figure 8 shows that the viscosity values of unaged and aged asphalt samples decreased progressively with incremental WPF addition; a variation that became more obvious with 6 and 9 wt.% of bio-based additive. This indicates that WPF cannot only lower advantageously the mixing and the compaction temperatures of the bitumen mixtures, and hence reducing greenhouse gas emissions, but also can more effectively reduce the aged asphalt viscosity (i.e., brittleness), which is vital in maintaining a proper balance of maltenes-to-asphaltenes ratio in the recovered binder. RTFO- and PAV-conditioned asphalt specimens possess relatively comparable viscosity values with 0 and 3 wt.% of rejuvenator. Using WPF-modified binders with viscosity lower than 3 Pa·s (< 3000 cP) can improve the fluidity of aged asphalt as well as the construction temperature and energy consumption of hot-mix asphalt (HMA). However, an extremely low viscosity would lead to the adhesion failure at the aggregate–bitumen interface and hence the deterioration of asphalt mixture performance.

### 3.6. Temperature Susceptibility

**Figure 9:**
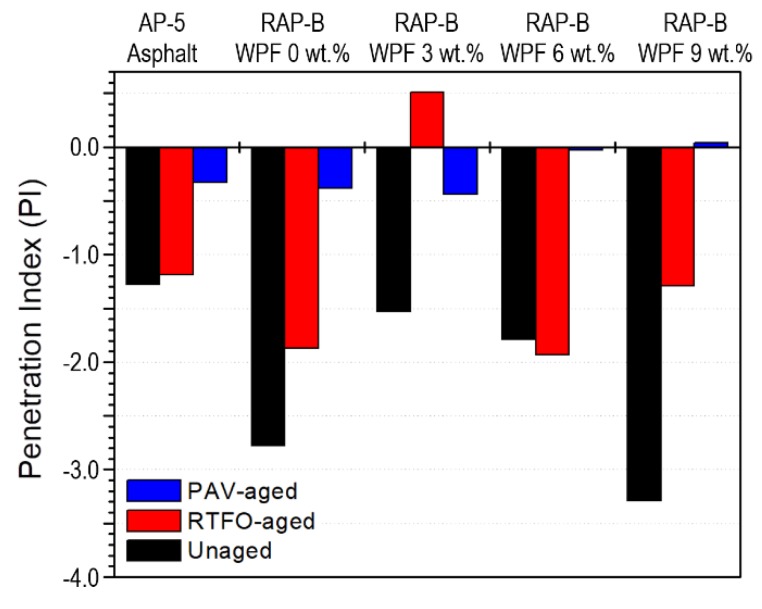
Effect of different concentrations (0, 3, 6, 9 wt.%) of WPF on the penetration index (PI) of RAP-B before and after RTFO and PAV aging.

**Figure 10:**
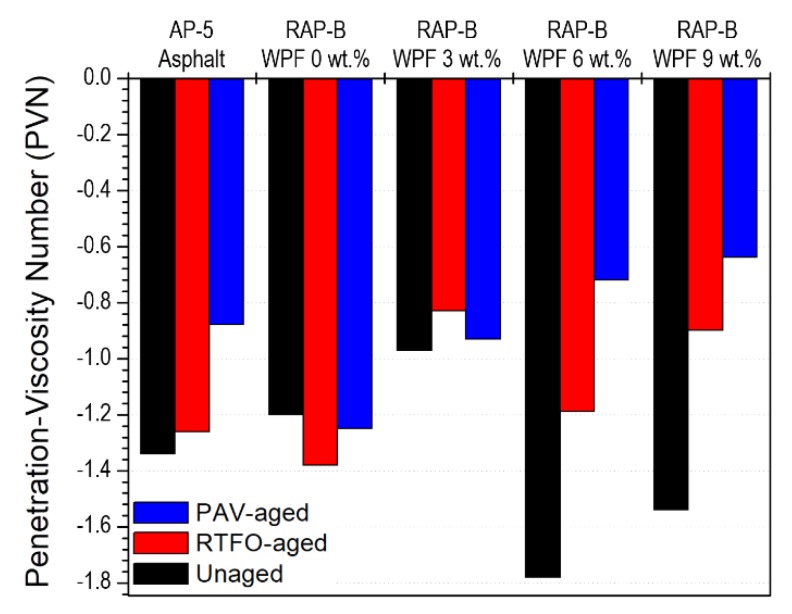
Effect of different concentrations (0, 3, 6, 9 wt.%) of WPF on the penetration-viscosity number (PVN) of RAP-B before and after RTFO and PAV aging.

The temperature susceptibility of unaged and aged bitumen samples containing different fractions (0, 3, 6, and 9 wt.%) of WPF were characterized by two approaches: penetration index (PI) and penetration viscosity number (PVN). Figure 9 demonstrates that the PI values are ranged between +0.51 and −3.29. Generally, good paving binders possess a PI value situated between +1 and −1 and if this value goes down −2, then high temperature susceptibility happens [36]. The unaged RAP-B enclosing 9 wt.% WPF exhibits the lowest PI value, whereas the unaged RAP-B modified with 3 wt.% WPF shows the highest PI. Hence, RAP-B WPF 9 wt.% sample is more susceptible to brittleness than other bituminous samples, resulting thereby in cracks in cold weather regions and severe rutting at high temperatures. Figure 10 indicates that all PVN values are extended between −0.64 and −1.78. Typically, most common paving binders have a PVN between +0.05 to −2.00 [36]. The unaged RAP-B containing 6 wt.% WPF displays the lowest PVN value, whilst the unaged RAP-B treated with 3 wt.% WPF exhibits the highest. Accordingly, RAP-B WPF 6 wt.% is the most temperature-susceptible binder and RAP-B WPF 3 wt.% is the least temperature-susceptible bitumen. Arguably, it can be said that 3 wt.% of bio-based rejuvenator can greatly enhance the temperature susceptibility properties of aged/oxidized bitumen in the range from 25 to 135 °C.

### 3.7. Dynamic Shear Rheometer (DSR) Test

#### 3.7.1. Rutting Resistance Factor (G*/sin δ)

In addition to the RAP-B, a Superpave performance graded binder, AP-5 asphalt PG 50–60, was used as a reference. To characterize their rutting performances, the untreated and WPF treated-RAP binders were subjected to DSR testing before and after short-term aged condition (RTFO). The rutting potential is assessed by the rutting resistance factor, denoted as G*/sin δ, of which the complex modulus (G*) and phase angle (δ) can be extracted directly from DSR testing. In general, to make the HMA less prone to rutting, a high G* value is desirable since it indicates a higher total resistance to deformation and, a lower δ value is desirable since it mirrors a more elastic component of the total deformation. In the Superpave system specifications, bitumen rutting is considered as a stress-controlled cyclic loading phenomenon. Considering the rutting as a stress-controlled, cyclic loading phenomenon, it can be admitted that rutting potential is inversely related to G*/sin δ. Therefore, minimum values are set on G*/sin δ. The diagnosis of the rutting factor of a given binder prior to long-term aging is pretty critical, since the aging process increases the stiffness of bitumen and its resistance to rutting. At high temperatures and a frequency of 10 rad s^−1^, the minimum required values of G*/sin δ are 1.00 kPa and 2.2 kPa for the unaged and RTFO-aged binders, respectively. These requirements are established to control and monitor the rutting of road pavements [27].

**Figure 11:**
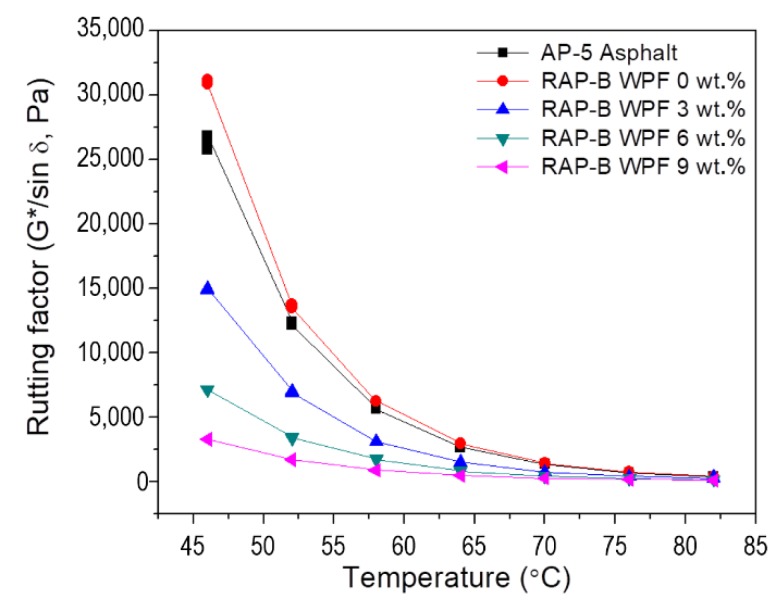
Variation of rutting factor (G*/sin δ) as function of temperature for unaged AP-5 asphalt and unaged RAP-B containing different concentrations (0, 3, 6, and 9 wt.%) of WPF.

**Figure 12:**
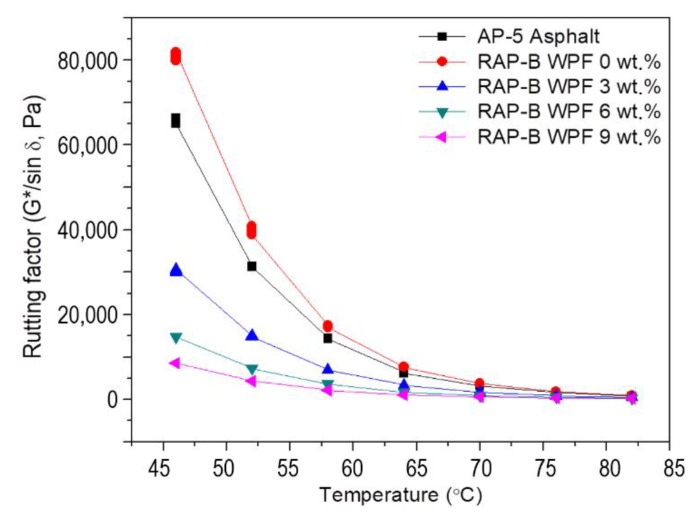
Variation of rutting factor (G*/sin δ) as function of temperature for RTFO-aged AP-5 asphalt and RTFO-aged RAP-B containing different concentrations (0, 3, 6, and 9 wt.%) of WPF.

In this work, the rutting potential of RTFO bitumen is determined by using DSR test. This test is equally applied on the unaged bitumen samples, because in some circumstances the RTFO may not reflect the actual aging of the binder during manufacturing and placing of hot-asphalt mixtures. Figure 11 and Figure 12 present the results of rutting resistance for unaged and short-term aged conditions obtained from DSR test, respectively. For RAP-B displays higher rutting resistance as compared to the reference binder AP-5 asphalt and WPF-modified asphalts (RAP-B WPF 3, 6, and 9 wt.%). It can be clearly noticed that the rutting resistance values were substantially decreased with the increase of the test temperatures ranging between 46 and 82 °C. It is also observed that the G*/sin δ value for the asphalt decreases as the rejuvenator content increases, which is deemed as an undesirable attribute at certain degree. Additionally, it can be seen that at a temperature of 51 °C and below, the pure asphalt as well as the WPF-modified asphalts fulfilled the minimum requirements (G*/sin δ > 1 kPa) of Superpave construction guidelines. The bitumen blended with 3, 6, and 9 wt.% WPF met the minimum requirements for rutting resistance below 64, 57, and 51 °C, respectively, and WPF-untreated bitumen met the minimum requirements below 70 °C.

The impact of WPF on rutting resistance factor of RTFO binders are shown in Figure 12. In reviewing this figure, it becomes evident that the rutting resistance factor of rejuvenated binders is strongly dependent upon test temperatures and WPF concentration. As illustrated in Figure 12, the rutting resistance parameter (G*/sin δ) decreases as the testing temperature and WPF fraction increase. These findings underscore the fact that rejuvenated binders that have an excessive incorporation of rejuvenator (especially 6 and 9 wt.%) would have a poor rutting resistance, which are in tune with the results of R&B softening point and viscosity (Figure 7 and Figure 8). On the basis of these results, at temperatures beyond 64, 58, and 57 °C, the asphalt samples treated with 3, 6, and 9 wt.% of WPF, respectively, are unable to resist rutting, whilst AP-5 asphalt and WPF-untreated RAP-B sample can resist until 70 °C. Accordingly, the unmodified binder—RAP-B—exhibits better resistance to rutting virtually at all temperatures as compared with the reference and modified samples. Similar trends were observed for unaged asphalt binders.

Conclusively, before and after RTFO, the asphalt cement comprising higher WPF doses exhibits the lowest rutting resistance, demonstrating thereby that extensive use of grease may alter dramatically the rutting performance of bitumen mixtures. Therefore, lower rejuvenator doses (less than 3 wt.%) may be effective in recovering the plastic deformation of aged bitumen (RAP-B).

#### 3.7.2. Fatigue Cracking Factor (G*⋅sin δ)

**Figure 13:**
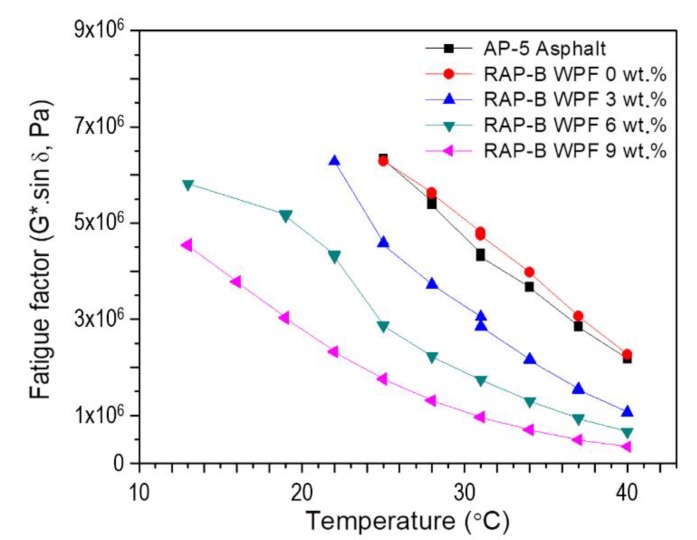
Variation of fatigue factor (G*⋅sin δ) as function of temperature for PAV-aged AP-5 asphalt and PAV-aged RAP-B containing different fractions (0, 3, 6, 9 wt.%) of WPF.

To determine the fatigue cracking performance of unmodified and WPF-modified asphalt specimens, the reference AP-5 asphalt (PG 50–60) and RAP-B underwent the dynamic shear rheometer (DSR) testing. The fatigue potential is assessed by the fatigue cracking factor, known as G*⋅sin δ. It is executed because one can see that under a strain-controlled condition, the work performed for fatigue is proportional to G*⋅sin δ, and hence, a lower value of G*⋅sin δ signifies a lower potential of fatigue cracking. This can be attributed to the fact that as the complex modulus (G*) decreases, the bitumen becomes less stiff (or brittle) and is capable to deform without generating large stresses and strains. Additionally, as the phase angle (δ) value decreases, the bitumen becomes more elastic and less viscoelastic and hence, can return to its original state without dissipating energy. In order to reduce the potential of fatigue cracking, a maximum limit on the value of G*⋅sin δ is defined in the Superpave specification. At intermediate temperatures and a frequency of 10 rad s^−1^, the maximum acceptable value of G*⋅sin δ is 5000 kPa [28]. This requirement is particularly designed to control and monitor the fatigue cracking of road pavements. The ability of a binder to dissipate stresses or relax is considered as a desirable feature in mitigating fatigue cracking. Since the bitumen becomes stiffer and brittle owing to aging during its service life and becomes more vulnerable to cracking, the standard DSR test is conducted on the PAV conditioned asphalt samples to determine the fatigue factor. The variation of the fatigue cracking resistance parameter with increasing WPF concentration in RAP-B at moderate temperatures (ranging between 13 and 40 °C) is shown in Figure 13. A close inspection of Figure 13 shows clearly there is a steady drop in G*⋅sin δ values as the rejuvenator content increases, indicating less shearing energy loss and higher fatigue cracking resistance. The different asphalt binders treated with 0, 3, 6, and 9 wt.% WPF satisfied the qualification of Superpave specifications at 31, 25, 19, and 13 °C, respectively. It can be concluded that WPF introduction into the RAP-B improves greatly the resistance potential to fatigue cracking at intermediate temperatures.

### 3.8. Performance Grade (PG) Test

**Figure 14:**
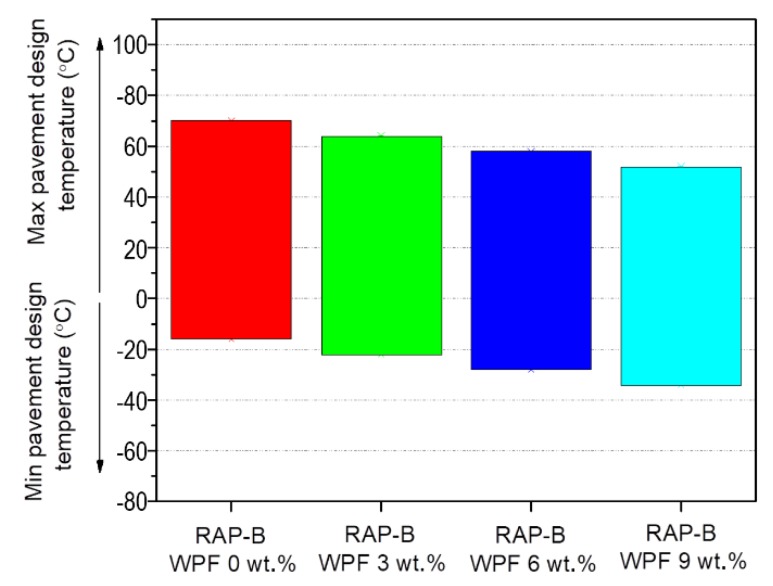
Effect of different concentrations (0, 3, 6, 9 wt.%) of WPF on the performance grade (PG) of RAP-B.

Figure 14 depicts the variation of performance grade (PG) of unmodified and RAP-B modified with different concentrations (3, 6, and 9 wt.%) of WPF, which is determined based on the dynamic shear rheometer data and Superpave performance classification system [43]. Referring to this figure, by increasing the WPF content, the performance of rejuvenated bitumen samples shows a substantial enhancement at low temperatures, insofar that the inferior limit of asphalts’ performance grade was dropped from −16 °C (RAP-B WPF 0 wt.%) to −34 °C (RAP-B WPF 9 wt.%). Each 3 wt.% of softening additive bumped low-temperature performance (i.e., critical temperature for thermal cracking) by +1 grade below −16 °C. Despite the proven positive impact of grease on the enhancement of PG at low temperatures, the bio-additive unfavorably altered the high-temperature performance (i.e., critical temperature without permanent deformation, rutting) causing decreases of up to one performance grade for each 3 wt.% WPF added. The recycling agent can permit greater flexibility and the ability to select an adequate dose that can specifically meet the climate and traffic conditions of the asphalt pavement being designed. It should also be stressed that dose selection does not ensure total or satisfactory pavement performance.

### 3.9. Thermogravimetric Analysis (TGA)

**Figure 15:**
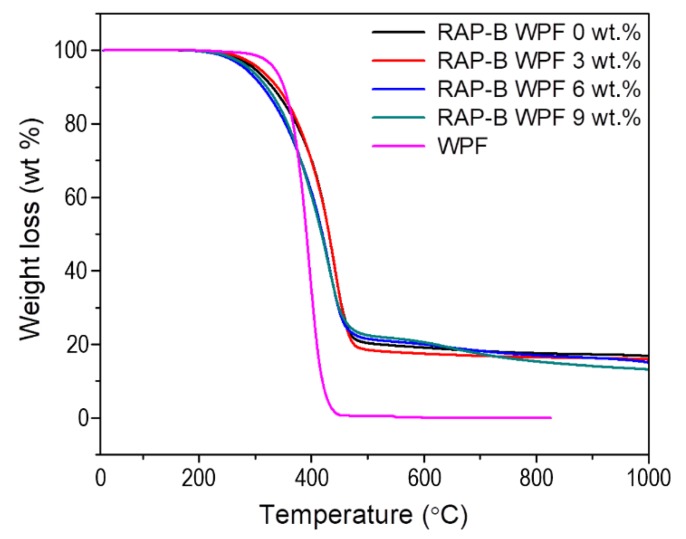
TGA thermograms of WPF, RAP-B, and WPF-modified RAP-Bs.

**Figure 16:**
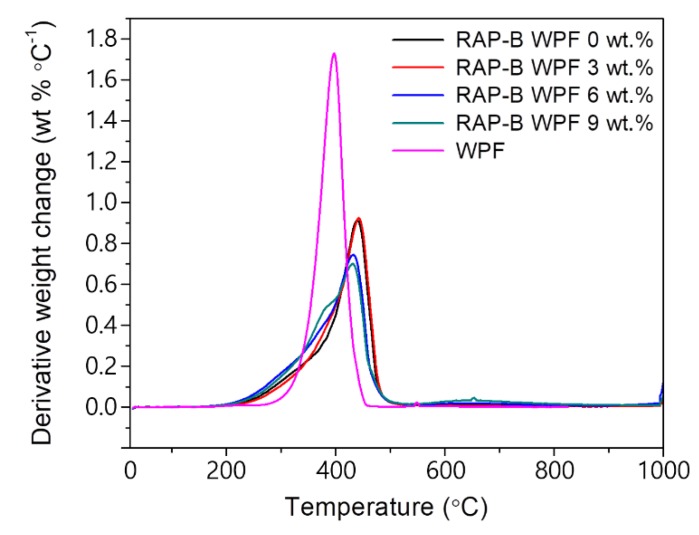
DTGA thermograms of WPF, RAP-B, and WPF-modified RAP-Bs.

Thermogravimetric analysis (TGA) was employed to monitor the weight changes in bituminous materials as a function of the temperature. Figure 15 and Figure 16 show the TGA and DTGA curves of WPF and RAP-B containing different fractions (0, 3, 6, and 9 wt.%) of WPF under N_2_ atmosphere, respectively. The TGA of bio-additive, WPF shows a plateau up to ~362.6 °C indicating an outstanding thermal stability. The decomposition of WPF occurs from 362.6 °C to 420.9 °C without residue and the maximum decomposition exhibits at 397.7 °C in DTGA. On the other hand, the TGA of RAP-B show the weight loss from ca. 250 °C up to ca. 440 °C remaining ca. 16.7 wt.% of residue. The DTGA of RAP-B shows two distinct region of weight loss of which one lies in ca. 250–376 °C and the other lies in ca. 376–466 °C. The former may correspond to the release of primary volatile small substances such as saturates and aromatics, and be derived from the rupture of some fragile bonds, the later to the release of volatiles derived from thermal decomposition of organics including resins and asphaltene. The weight loss in the region of 466–1000 °C is very small remaining ca. 16.7 wt.% of char formed [44].

The different parameters obtained from TGA/DTGA curves (Figure 15 and Figure 16) are summarized in Table 3. As can be seen, the thermal stability of asphalt blends was slightly altered only after the addition of 6 and 9 wt.% WPF; whereas 3 wt.% of grease enhanced it barely. This fact could be probably attributed to the high unsaturated fatty acid content which made the asphalt binder more susceptible to an insignificant thermal deterioration. Overall, it can be assumed that the animal fat-based rejuvenator does not seem to adversely impact the thermal properties of bitumen mixtures and can effectively be used to reactivate the old binder of reclaimed asphalt pavement at normal operational temperatures (T_max_ of HMA mixing and manufacturing < 200 °C).

### 3.10. Differential Scanning Calorimetry (DSC)

**Figure 17:**
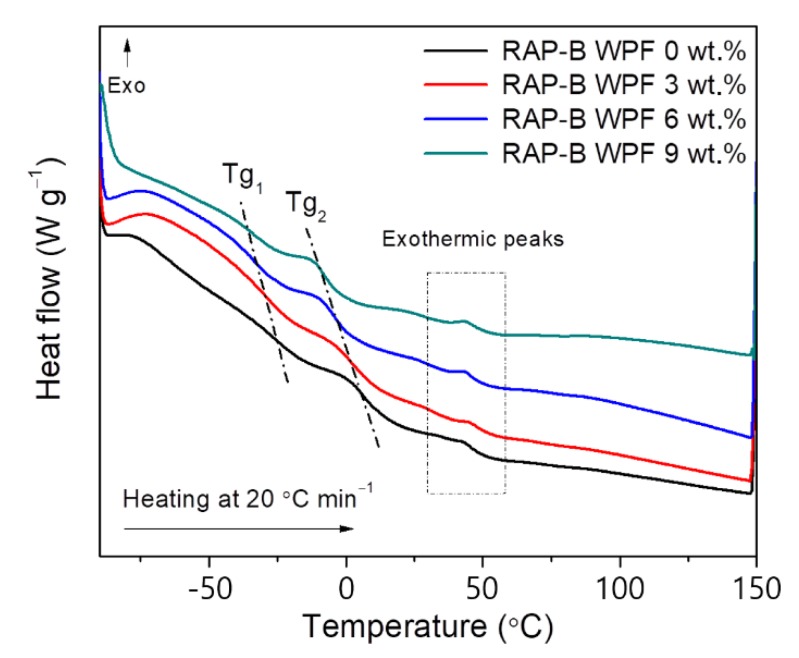
DSC thermograms of RAP-B and WPF-modified RAP-Bs.

Differential scanning calorimetry (DSC) technique was used to in-depth study the thermal behavior of grease-binder mixtures. Figure 17 shows that the DSC thermograms of RAP-B and asphalt treated with different concentrations (3, 6, and 9 wt.%) of WPF demonstrates the presence of two different glass transition temperatures (Tg). Tg_1_, is located between −34 °C and −25 °C and Tg_2_, is situated between −7 °C and 9 °C. Various exothermic peaks, can be readily detected between 25 and 50 °C, related to crystallizable elements (e.g., paraffins and naphthene aromatics) or to melting of saturates present within the bitumen matrix [45].

As Figure 17 and Table 4 show, with increasing WPF content up to 9 wt.%, Tg_1_ and Tg_2_ gradually decreased from −25 °C to −34 °C and from 8 °C to −6 °C, respectively. Tg_1_ arises from the maltene phase and it is vital for the low temperature properties of asphalt binders. The second one, Tg_2_, stems from the maltene–asphaltene interfacial region of mixed composition likely rich in resins (the so-called “interphase”) [45]. Below Tg, bitumen is being rigid and brittle like glass, affecting thereby the tensile strength as well as the fatigue performance of binder or resultant mixtures [46]. These results support that the bio-based rejuvenator, WPF, greatly mitigates the adverse effect of aging on embrittlement and durability, improving binder’s flexibility and performance at low temperatures.

**Figure 18:**
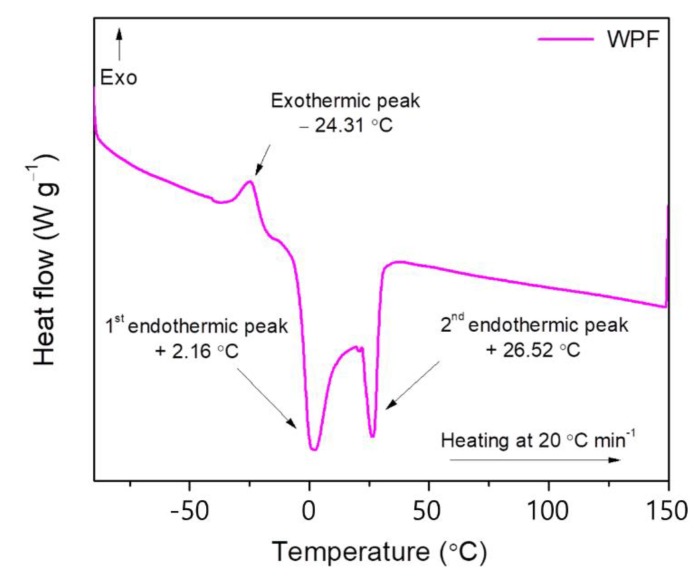
DSC thermogram of waste pig fat (WPF).

The DSC curve of WPF in Figure 18 exhibits a large melting range (0 °C < T_endo_ < 30 °C) with a single crystallization peak at −24.3 °C. The more saturated the TAG (triacylglycerol), the higher the melting temperature; and conversely, the less saturated TAG, the lower the melting temperature. Thus, the first major peak at around +2 °C is caused mostly by the unsaturated FA (fatty acids) and TAG and the second peak around +26 °C is caused chiefly by the saturated FA and TAG existing in the pig fats. It can be seen that the first major peak is larger than the second endothermic peak. The WPF contains apparently more unsaturated fatty acids than saturated ones and this is in accordance with the fat composition findings (Table 1). There is also one minor exothermic peak at around −24 °C. It has been reported that the intensity of this peak varies with the nature of lard, the feeding type, and the variety of fatty acid composition. The saturated FA and TAG crystallize at high temperature, whereas the unsaturated FA and TAG crystallize at low temperatures [47].

## 4. Conclusions

WPF as a highly effective mixture of fatty acids can increase the durability of the reclaimed asphalt pavement, by restoring its original balance of chemical constituents, which are compromised during aging process. In essence, this outstanding thermally stable bio-product can also lower the binder mixing and compaction temperatures, thus yielding considerable energy savings. WPF is characterized by its ease of use and good compatibility with aged asphalt. In addition to shifting the PG grade to low-temperatures, the bio-modifier can revitalize and rehabilitate the reclaimed asphalt pavement binder, allowing for more versatility in mix designs. It can also assist in adjusting the physico-rheological properties of recovered binder by reducing viscosity and softening point values and increasing penetration values and corresponding DSR values, extending thereby the pavement’s life cycle. Improvements include, but are not limited to, good low-temperature performance, restored temperature sensitivity, good binder–aggregate adhesion/cohesion, and superior resistance to fatigue cracking, etc. Despite the benefits of WPF as a promising, safe, and ecofriendly bio-rejuvenator, some remaining challenges regarding its impact on rutting and high-temperature PG performance should be addressed.

## Figures and Tables

**Table 1:** Physicochemical characteristics of waste pig fat (WPF).

Property	Value
Acid value (mg of KOH/g)	0.71
Iodine value (g of I_2_/ 100 g)	67
Moisture content (wt.%)	0.03
Fatty acid composition (wt.%)	
Saturated fats	39.20
Unsaturated fats	
Total monounsaturated	45.10
Total polyunsaturated	11.20

**Table 2:** Physicochemical properties of RAP-B.

**Elemental analysis**	Ave	SD
C (carbon)	85.16 wt.%	0.40
H (hydrogen)	9.40 wt.%	0.24
N (nitrogen)	0.94 wt.%	0.05
S (sulfur)	3.80 wt.%	0.21
O (oxygen)	0.70 wt.%	0.08
**SARA Generic fractions**	Ave	SD
Saturates	5.09 wt.%	0.41
Aromatics	12.94 wt.%	0.71
Resins	38.53 wt.%	2.35
Asphaltenes	43.44 wt.%	2.24
**Physical properties**	Ave	SD
Penetration at 25 °C, 0.1 mm	60.00	1.0
Softening point	42.9 °C	0.5
Viscosity at 135 °C	309 cP	11

**Table 3:** TGA and DTGA data of WPF, RAP-B, and WPF-modified RAP-Bs.

Sample	TGA/DTGA (°C)		ΔW (wt.%)
	ΔT_dec_	T_max_	
WPF	362.6–420.9	397.7	0
RAP-B WPF 0 wt.%	376.0–466.0	439.8	16.7
RAP-B WPF 3 wt.%	376.7–467.2	442.4	15.8
RAP-B WPF 6 wt.%	351.3–458.0	432.0	14.6
RAP-B WPF 9 wt.%	345.3–464.6	431.3	12.9

ΔT_dec_, the temperature range of decomposition; T_max_, maximum decomposition temperature (°C); ΔW, carbonaceous residue at 1000 °C.

**Table 4:** DSC data of RAP-B and RAP-B blended with 3, 6, and 9 wt.% WPF.

Sample	Tg_1_ (°C)	ΔCp_1_ (J/g⋅°C)	Tg_2_ (°C)	ΔCp_2_ (J/g⋅°C)
RAP-B WPF 0 wt.%	–25.3	0.238	8.1	0.277
RAP-B WPF 3 wt.%	–29.7	0.311	3.8	0.277
RAP-B WPF 6 wt.%	–33.2	0.285	–3.4	0.262
RAP-B WPF 9 wt.%	–33.8	0.208	–6.1	0.257

## References

[1] AbuQtaish L., Nazzal M.D., Kaya S., Kim S.S., Abbas A., Abu Hassan Y. (2018). AFM-Based Approach to Study Blending between RAP and Virgin Asphalt Binders. J. Mater. Civil. Eng..

[2] Petersen J.C., Glaser R. (2011). Asphalt Oxidation Mechanisms and the Role of Oxidation Products on Age Hardening Revisited. Road Mater. Pavement.

[3] Sirin O., Paul D.K., Kassem E. (2018). State of the Art Study on Aging of Asphalt Mixtures and Use of Antioxidant Additives. Adv. Civ. Eng..

[4] Delgadillo R., Bahia H.U. (2008). Effects of Temperature and Pressure on Hot Mixed Asphalt Compaction: Field and Laboratory Study. J. Mater. Civil. Eng..

[5] Mogawer W.S., Austerman A., Roque R., Underwood S., Mohammad L., Zou J. (2015). Ageing and rejuvenators: Evaluating their impact on high RAP mixtures fatigue cracking characteristics using advanced mechanistic models and testing methods. Road Mater. Pavement.

[6] McDaniel R.S., Shah A., Huber G.A., Copeland A. (2012). Effects of reclaimed asphalt pavement content and virgin binder grade on properties of plant produced mixtures. Road Mater. Pavement.

[7] Mogawer W.S., Booshehrian A., Vahidi S., Austerman A.J. (2013). Evaluating the effect of rejuvenators on the degree of blending and performance of high RAP, RAS, RAP/RAS mixtures. Road Mater. Pavement.

[8] Zaumanis M., Mallick R.B., Poulikakos L., Frank R. (2014). Influence of six rejuvenators on the performance properties of Reclaimed Asphalt Pavement (RAP) binder and 100% recycled asphalt mixtures. Constr. Build. Mater..

[9] Tran N., Taylor A., Turner P., Holmes C., Porot L. (2016). Effect of rejuvenator on performance characteristics of high RAP mixture. Road Mater. Pavement.

[10] Kuang D., Jiao Y., Ye Z., Lu Z., Chen H., Yu J., Liu N. (2018). Diffusibility Enhancement of Rejuvenator by Epoxidized Soybean Oil and Its influence on the Performance of Recycled Hot Mix Asphalt Mixtures. Materials.

[11] Lin J., Guo P., Xie J., Wu S., Chen M. (2013). Effect of Rejuvenator Sealer Materials on the Properties of Aged Asphalt Binder. J. Mater. Civ. Eng..

[12] Xie Z., Tran N., Julian G., Taylor A., Blackburn L.D. (2017). Performance of Asphalt Mixtures with High Recycled Contents Using Rejuvenators and Warm-Mix Additive: Field and Lab Experiments. J. Mater. Civ. Eng..

[13] Zaumanis M., Mallick R.B., Frank R. (2013). Evaluation of Rejuvenator’s Effectiveness with Conventional Mix Testing for 100% Reclaimed Asphalt Pavement Mixtures. Transp. Res. Record.

[14] Mamun A.A., Al-Abdul Wahhab H.I. (2018). Evaluation of Waste Engine Oil-Rejuvenated Asphalt Concrete Mixtures with High RAP Content. Adv. Civ. Eng..

[15] Wallace T., Gibbons D., O’Dwyer M., Curran T.P. (2017). International evolution of fat, oil and grease (FOG) waste management—A review. J. Environ. Manage..

[16] Ministry of Environment (2014). 2013 Generation and Disposal of Waste in South Korea.

[17] El-Adawy M., Ibrahim A., El-Kassaby M.M. (2013). An Experimental Evaluation of using Waste Cooking Oil Biodiesel in a Diesel Engine. Energy Technol..

[18] Murayama T., Fujiwara Y., Noto T. (2000). Evaluating waste vegetable oils as diesel fuel. P. I. Mech. Eng. D. J. Aut..

[19] Ndiaye M., Arhaliass A., Legrand J., Roelens G., Kerihuel A. (2020). Reuse of waste animal fat in biodiesel: Biorefining heavily-degraded contaminant-rich waste animal fat and formulation as diesel fuel additive. Renew. Energ..

[20] Maharaj R., Ramjattan-Harry V., Mohamed N. (2015). Rutting and Fatigue Cracking Resistance of Waste Cooking Oil Modified Trinidad Asphaltic Materials. Sci. World J..

[21] Zargar M., Ahmadinia E., Asli H., Karim M.R. (2012). Investigation of the possibility of using waste cooking oil as a rejuvenating agent for aged bitumen. J. Hazard. Mater..

[22] Bailey H.K., Philips P. (2010). Asphalt Rejuvenation. U.S. Patent.

[23] Ji J., Yao H., Suo Z., You Z., Li H., Xu S., Sun L. (2017). Effectiveness of Vegetable Oils as Rejuvenators for Aged Asphalt Binders. J. Mater. Civil. Eng..

[24] American Society of Testing Materials (ASTM) International (2015). ASTM D946/D946M-15, Standard Specification for Penetration-Graded Asphalt Binder for Use in Pavement Construction.

[25] American Association of State Highway and Transportation Officials (2014). AASHTO T 164, Standard Method of Test for Quantitative Extraction of Asphalt Binder from Hot-Mix Asphalt (HMA).

[26] American Society of Testing Materials (ASTM) International (2012). ASTM D5404 / D5404M-12, Standard Practice for Recovery of Asphalt from Solution Using the Rotary Evaporator.

[27] American Society of Testing Materials (ASTM) International (2019). ASTM D8272-19, Standard Test Method for Effect of Heat and Air on a Moving Film of Asphalt (Rolling Thin-Film Oven Test).

[28] American Society of Testing Materials (ASTM) International (2013). ASTM D6521-13, Standard Practice for Accelerated Aging of Asphalt Binder Using a Pressurized Aging Vessel (PAV).

[29] American Association of State Highway and Transportation Officials (2015). AASHTO T 30, Standard Method of Test for Mechanical Analysis of Extracted Aggregates.

[30] Loeber L., Sutton O., Morel J., Valleton J.M., Muller G. (1996). New direct observations of asphalts and asphalt binders by scanning electron microscopy and atomic force microscopy. J. Microsc..

[31] Mazumder M., Ahmed R., Ali A.W., Lee S.J. (2018). SEM and ESEM techniques used for analysis of asphalt binder and mixture: A state of the art review. Constr. Build. Mater..

[32] Butt H.-J., Jaschke M. (1995). Calculation of thermal noise in atomic force microscopy. Nanotechnology.

[33] American Society of Testing Materials (ASTM) International (2013). ASTM D5/D5M-13, Standard Test Method for Penetration of Bituminous Materials.

[34] American Society of Testing Materials (ASTM) International (2014). ASTM D36/D36M-14e1, Standard Test Method for Softening Point of Bitumen (Ring-and-ball Apparatus).

[35] American Society of Testing Materials (ASTM) International (2015). ASTM D4402/D4402M-15, Standard Test Method for Viscosity Determination of Asphalt at Elevated Temperatures Using a Rotational Viscometer.

[36] Roberts F.L., Kandhal P.S., Brown E.R., Lee D.Y., Kennedy T.Y. (1991). Hot Mix Asphalt Materials, Mixture Design and Construction.

[37] American Society of Testing Materials (ASTM) International (2015). ASTM D7175-15, Standard Test Method for Determining the Rheological Properties of Asphalt Binder Using a Dynamic Shear Rheometer.

[38] Cordella C., Moussa I., Martel A.C., Sbirrazzuoli N., Lizzani-Cuvelier L. (2002). Recent Developments in Food Characterization and Adulteration Detection: Technique-Oriented Perspectives. J. Agr. Food Chem..

[39] Coates J. (2000). Interpretation of Infrared Spectra, A Practical Approach. Encyclopedia of Analytical Chemistry, Meyers, R.A., Eds..

[40] Masson J.F., Leblond V., Margeson J. (2006). Bitumen morphologies by phase-detection atomic force microscopy. J. Microsc. Oxf..

[41] Pauli A.T., Grimes R.W., Beemer A.G., Turner T.F., Branthaver J.F. (2011). Morphology of asphalts, asphalt fractions and model wax-doped asphalts studied by atomic force microscopy. Int. J. Pavement Eng..

[42] Das P.K., Kringos N., Wallqvist V., Birgisson B. (2013). Micromechanical investigation of phase separation in bitumen by combining atomic force microscopy with differential scanning calorimetry results. Road Mater. Pavement.

[43] American Society of Testing Materials (ASTM) International (2016). ASTM D6373-16, Standard Specification for Performance Graded Asphalt Binder.

[44] Jing-Song G., Wei-Biao F., Bei-Jing Z. (2003). A study on the pyrolysis of asphalt. Fuel.

[45] Nciri N., Kim N., Cho N. (2017). New insights into the effects of styrene-butadiene-styrene polymer modifier in the structure, properties, and performance of asphalt binder: The case of AP-5 asphalt and solvent deasphalting pitch. Mater. Chem. Phys..

[46] Elseifi M., Mohammad L.N., Glover I., Negulescu I., Daly W.H., Abadie C. (2010). Relationship between Molecular Compositions and Rheological Properties of Neat Asphalt Binder at Low and Intermediate Temperatures. J. Mater. Civ. Eng..

[47] Azir M., Abbasiliasi S., Ibrahim T.A.T., Manaf Y.N.A., Sazili A.Q., Mustafa S. (2017). Detection of Lard in Cocoa Butter—Its Fatty Acid Composition, Triacylglycerol Profiles, and Thermal Characteristics. Foods.

